# High Inorganic Phosphate Intake Promotes Tumorigenesis at Early Stages in a Mouse Model of Lung Cancer

**DOI:** 10.1371/journal.pone.0135582

**Published:** 2015-08-18

**Authors:** Somin Lee, Ji-Eun Kim, Seong-Ho Hong, Ah-Young Lee, Eun-Jung Park, Hwi Won Seo, Chanhee Chae, Philip Doble, David Bishop, Myung-Haing Cho

**Affiliations:** 1 Laboratory of Toxicology, BK21 PLUSProgram for Creative Veterinary Science Research, Research Institute for Veterinary Science and College of Veterinary Medicine, Seoul National University, Seoul, 151–742, Korea; 2 Graduate School of Convergence Science and Technology, Seoul National University, Suwon, 443–270, Korea; 3 Department of Molecular Science and Technology, Ajou University, Suwon, 443–749, Korea; 4 Laboratory of Pathology, College of Veterinary Medicine, Seoul National University, Seoul, 151–742, Korea; 5 Elemental Bio-imaging Facility, Department of Chemistry and Forensic Science, University of Technology, Sydney, Australia; 6 Graduate Group of Tumor Biology, Seoul National University, Seoul, 151–742, Korea; 7 Advanced Institute of Convergence Technology, Seoul National University, Suwon, 443–270, Korea; Sun Yat-sen University Cancer Center, CHINA

## Abstract

Inorganic phosphate (Pi) is required by all living organisms for the development of organs such as bone, muscle, brain, and lungs, regulating the expression of several critical genes as well as signal transduction. However, little is known about the effects of prolonged dietary Pi consumption on lung cancer progression. This study investigated the effects of a high-phosphate diet (HPD) in a mouse model of adenocarcinoma. K-ras^LA1^ mice were fed a normal diet (0.3% Pi) or an HPD (1% Pi) for 1, 2, or 4 months. Mice were then sacrificed and subjected to inductively coupled plasma mass/optical emission spectrometry and laser ablation inductively coupled plasma mass-spectrometry analyses, western blot analysis, histopathological, immunohistochemical, and immunocytochemical analyses to evaluate tumor formation and progression (including cell proliferation, angiogenesis, and apoptosis), changes in ion levels and metabolism, autophagy, epithelial-to-mesenchymal transition, and protein translation in the lungs. An HPD accelerated tumorigenesis, as evidenced by increased adenoma and adenocarcinoma rates as well as tumor size. However, after 4 months of the HPD, cell proliferation was arrested, and marked increases in liver and lung ion levels and in energy production via the tricarboxylic acid cycle in the liver were observed, which were accompanied by increased autophagy and decreased angiogenesis and apoptosis. These results indicate that an HPD initially promotes but later inhibits lung cancer progression because of metabolic adaptation leading to tumor cell quiescence. Moreover, the results suggest that carefully regulated Pi consumption are effective in lung cancer prevention.

## Introduction

Inorganic phosphate (Pi) is required by all living organisms for diverse cellular functions—including mineral metabolism and energy production from membrane phospholipids and nucleotides—and as a substrate for phosphorylated intermediates in cell signaling [1]. Pi can be acquired passively through food intake or actively via cellular metabolism. It is often used as a food additive not for health reasons, but for functions such as maintaining food color and flavor, acid buffering, leavening, texture stabilization, and prolongation of shelf life. A range of food products contain Pi, including cashews, almonds, bacon, canned fish, evaporated milk, processed cheeses, baking powder, baker’s yeast, eggs, lentils, and isotonic and cola beverages [2]. Human Pi consumption is increasing owing to the availability of these foods; studies indicate that the use of Pi in food additives has steadily increased while Pi intake has risen by nearly 17% in recent decades [3].

The latest reports suggest that abnormal Pi intake is closely correlated with the occurrence of various types of cancer [4]. Pi also plays a crucial role in gene regulation during the cell cycle as well as in signal transduction and transcriptional control in vitro [5]. In addition, a high-phosphate diet (HPD) was shown to promote lung tumorigenesis through altered Akt signaling [6]. However, there have been no studies to date evaluating homeostatic maintenance in the lungs in relation to an HPD or the role of Pi in metabolic adaptation and cancer progression, which involves autophagy, apoptosis, ion level regulation, and adenosine triphosphate (ATP) synthesis via the tricarboxylic acid (TCA) cycle. The present study investigated the effects of high Pi intake on lung cancer progression and the underlying mechanisms in K-ras^LA1^ mice, in which constitutive activation of the K-ras oncogene leads to the development of lung adenocarcinoma. The results indicate that an HPD initially promotes but later inhibits lung cancer progression as a result of metabolic adaptation leading to tumor cell quiescence.

## Materials and Methods

### Animals and diet

Methods used in this study were approved by the Animal Care and Use Committee at Seoul National University (SNU-120904-3-2). Experiments were conducted on 5-week-old male K-ras^LA1^ mice obtained from the Human Cancer Consortium of the National Cancer Institute (Frederick, MD, USA). Animals were maintained in a laboratory facility at a standard temperature of 23°C ± 2°C and a relative humidity of 50% ± 10% under a 12:12 h light/dark cycle. A total of 36 mice were randomly allocated to six groups of six mice each; half of the mice (1, 2, and 4 month groups) were fed a standard American Institute of Nutrition (AIN) 93-based diet containing 0.3% Pi (normal diet, ND), while the other half (1, 2, and 4 month groups) were given the same diet supplemented with 1.0% Pi (HPD). The composition of the modified AIN-93G purified rodent diet [7] is shown in Table 1. Body weight, as well as the amount of pellet and volume of water consumed, was measured once a week. After 1, 2, or 4 months on the diet, mice were anesthetized with 15 mg/kg Zoletil (Laboratories Virbac, Carros, France) and 3 mg/kg xylazine (Laboratories Calier, Barcelona, Spain), and transcardial perfusion was carried out. During the autopsy, the brain, thymus, heart, lung, liver, spleen, kidney, and testis were removed. Tumors on the entire lung surface were counted, and lesion diameters were measured with digital calipers under a microscope as previously described [8].

**Table 1:** Composition of normal and high phosphate-modified AIN-93G rodent diets.

Ingredient	Normal diet (0.3% Pi)	High phosphate diet (1% Pi)
Casein, g	200	200
l-cysteine, g	3	3
Corn starch, g	397.486	397.486
Maltodextrin, g	132	132
Sucrose, g	100	100
Soybean oil, g	70	70
Cellulose (fiber), g	53.3	36.1
94047VM, AIN-93-VX, g	10	10
Choline bitartrate, g	2.5	2.5
t-Butylhydroquinone (antioxidant), g	0.014	0.014
AIN-93 mineral mixture (excluding Ca, P), g	13.4	13.4
Calcium carbonate (CaCO_3_), g	0	0
Calcium phosphate monobasic, g	3	31.5
Sodium phosphate monobasic, g	4	4

### Histopathological examination, immunohistochemistry, and immunocytochemistry

Tissue samples were fixed in 10% buffered formalin, embedded in paraffin, and cut at a thickness of 5 μm, with sections collected on charged glass slides (Fisher Scientific, Pittsburgh, PA, USA). For histopathological analysis, sections were deparaffinized in xylene and rehydrated through a graded series of alcohol, then stained with hematoxylin and eosin (H&E; Sigma-Aldrich, St Louis, MO, USA). For immunohistochemistry, sections were deparaffinized in xylene and rehydrated through a graded series of alcohol, washed, and then incubated in 3% hydrogen peroxide (AppliChem, Darmstadt, Germany) for 30 min to quench endogenous peroxidase activity, and then washed in phosphate-buffered saline (PBS) and incubated with 3% bovine serum albumin in Tris-/Tween-buffered saline for 1 h at room temperature to block nonspecific binding. Primary antibodies against proliferating cell nuclear antigen (PCNA) (PC10; Santa Cruz Biotechnology, Santa Cruz, CA, USA) and caspase-3 (#9662S; Abcam, Cambridge, MA, USA) were applied to sections at 1:200 and 1:1000 dilutions, respectively, overnight at 4°C. The following day, sections were washed and incubated with horseradish peroxidase-conjugated secondary antibodies (1:50) for 1 h at room temperature. After washing, sections were counterstained with Mayer’s hematoxylin (DakoCytomation, Carpinteria, CA, USA) and washed with xylene, and mounted using Permount (Fisher Scientific). Slides were viewed under a light microscope (Carl Zeiss, Thornwood, NY, USA). For immunocytochemistry, nuclei were stained with 1 μg/ml DAPI after blocking and washing three times with PBS. An antibody against microtubule-associated protein 1A/1B light chain (LC)3 was applied at 1:400 dilution overnight at 4°C. Sections were rinsed, incubated with fluorescein isothiocyanate-conjugated secondary antibody, and then mounted onto glass slides using Faramount aqueous mounting medium (DakoCytomation). Staining intensity was assessed by counting the number of positive cells in randomly selected fields using In Studio version 3.01 software (Pixera, San Jose, CA, USA).

### Western blot analysis

Total protein concentration in homogenized lung samples was determined using the Bio-Rad Protein Assay reagent (Bio-Rad, Hercules, CA, USA). Western blotting was performed according to a previously described procedure [9] using antibodies against the following proteins: acetyl coenzyme (Co)A (#3676), phosphorylated (p)-acetyl CoA (#3661), LC3 (#2275), autophagy protein (ATG)5 (#2630), phosphorylated (p)-p70 S6 Kinase^Thr389^(#9205), 4EBP1 (#9644S), and p-Akt^Thr308^ (#2965) (all from Cell Signaling Technology, Danvers, MA, USA); succinate dehydrogenase complex, subunit A (SDHA) (ab14715) and cytochrome c oxidase subunit (COX)-IV (ab33985) (both from Abcam); cytochrome c (A-8), Rieske (A-5), B-cell lymphoma (Bcl)-2 (C-2), Bcl-2-associated death promoter (Bad) (C-7), Bcl-2-associated X protein (Bax) (B-9), eIF4E (P-2), fibroblast growth factor (FGF)-2 (147), proliferating cell nuclear antigen (PCNA) (PC10), N-cadherin, and actin (I-19) (all from Santa Cruz Biotechnology); and glyceraldehyde-3-phosphate dehydrogenase (LF-PA0212; AbFrontier, Seoul, Korea). Protein bands were detected using the LAS-3000 luminescent image analyzer (Fujifilm, Tokyo, Japan) and quantified using Multi Gauge version 2.02 software (Fujifilm).

### qRT-PCR analysis

qRT-PCR analysis was conducted by isolating RNA using total RNA was isolated using the QuickGene RNA kit (Fujifilm’s Life Science System, Tokyo, Japan). Complementary DNA was produced with the SuPrimeScript RT Premix (GeNet Bio, Cheonan, Korea). The reactions were set up in triplicate with the Prime Q-Mastermix (GeNet Bio) and run on CFX96T Real-time System (Bio-Rad, Hercules, CA, USA). The gene expressions are presented as the expression relative to Actin. The sequences of the primers that were used for the qPCR were as follows mouse E-cadherin, forward (5’-TCGAGGAAATCTGCATTCATACTC-3’), reverse (5’-TCTCCGCTGCCATTTCTAAC-3’), mouse β-actin, forward (5’- TTTCCAGCCTTCCTTCTTGGGTATG-3’), and reverse (5’- CACTGTGTTGGCATAGAGGTCTTTAC-3’).

### Detection of trace elements in tissue

Laser ablation inductively coupled plasma-mass spectrometry (LA-ICP-MS) was performed using a New Wave Research UP-213 laser ablation system (Kenelec Technologies, New South Wales, Australia) equipped with a neodymium-doped yttrium aluminum garnet laser emitting a nanosecond laser pulse in the fifth harmonic with a wavelength of 213 nm. The laser was connected to a 7500ce ICP-MS (Agilent Technologies, Palo Alto, CA, USA) by Tygon tubing. Details of the analytical methods have been previously published [10]. Briefly, the laser beam was rastered along the sample surface in a straight line. Data were acquired with a laser spot size of 65 μm, laser scan speed of 20 μm s^−1^, and ICP-MS total integration time of 0.325 s. For ICP-optical emission spectrometry (ICP-OES) elemental analysis, tissue samples were digested in sulfuric acid and hydrogen peroxide. All chemical reagents were of analytical grade. Water (resistivity of 18.2 MΩ) was de-ionized with a Milli-Q system (Millipore, Bedford, MA, USA). Nitric acid (65% v/v) and sulfuric acid (95%–97% v/v) (both from Samchun Chemical Co., Seoul, Korea) were used for sample preparation. Acid digestion of tissue samples diluted with Milli-Q water using an acid concentration of 0.2% was carried out at 70°C for 24 h followed by hydrogen peroxide (70°C) digestion overnight. Concentrations of elemental Mn, Fe, Cu, and Zn were quantified by ICP-MS and concentrations of elemental Na, Ca, S, and P were measured by ICP-OES. Operational conditions for both procedures are shown in Table 2.

**Table 2:** ICP-MS and ICP-OES operational conditions.

Parameter	ICP-MS	ICP-OES
Model / Manufacturer	7700 / Agilent, Japan	5300DV / PerkinElmer Optima
Radio frequency generator power, W	1550	1300
Argon gas flow rate		
Plasma, l/min	15	15
Carrier, l/min	0.8	0.5
Makeup, l/min	0.35	0.65
Collision gas (He), ml/min	4	1
Replicates	3	3
Isotopes monitored	Mn, Zn, Cu, Fe	Na, Ca, S, P

### Statistical analysis

The Student’s *t*-test was used to evaluate differences between two groups (GraphPad Software, San Diego, CA, USA). Asterisks (*) indicated statistically significant differences to the ND.

## Results

### High Pi intake accelerates tumorigenesis at early stages in a lung cancer model

**Fig 1:**
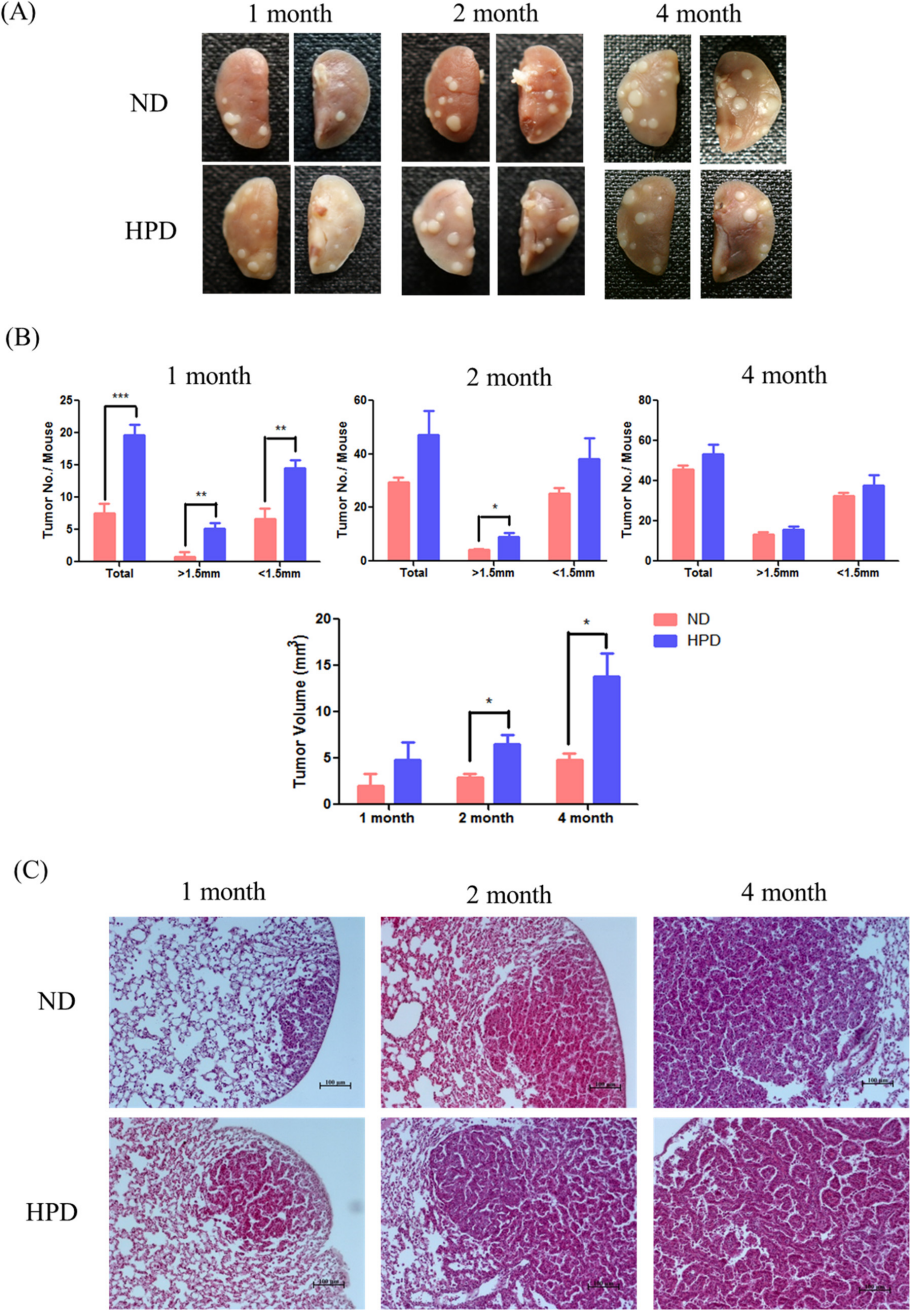
Tumor incidence in the lungs of K-ras^LA1^ mice provided with an ND or HPD. Mice (n = 6 per group) were fed an ND (0.3% Pi) or HPD (1% Pi) for 1, 2, or 4 months. (A) Adenocarcinoma and adenoma in the lung tissue. (B) Total number of tumors, number of tumors with diameter >1.5 mm, and tumor volume in ND- and HPD-fed mice. The results are mean ± standard deviation (SD) of six independent measurements. Error bar represent SD. *p < 0.05; **p < 0.01; ***p < 0.001. (C) Tumors on the lungs of ND- and HPD-fed mice, as visualized by H&E staining. Scale bar: 100 μm.

To investigate the effect of high Pi intake on lung cancer cell growth, tumors on the lung surface were measured in HPD- and ND-fed mice (Fig 1A). All HPD-fed mice exhibited progressive adenocarcinoma and adenoma, as observed by H&E staining (Fig 1C). A high Pi consumption induced the development of lung cancer over periods of 1, 2, and 4 months (Tables 3 and 4). The number and size of tumor nodules were higher in the HPD than in the ND group at 1 and 2 months (P < 0.05), although the difference between the groups was not statistically significant at 4 months (Fig 1B). The tumor volume was also higher in mice consuming an HPD than in ND mice, but only in the 2- and 4-month groups (Fig 1B). A histopathological examination revealed an approximately 2-fold higher incidence of adenocarcinoma and adenoma in the 1- and 2-month HPD groups than in their ND counterparts. However, in the 4-month HPD group, the incidences of adenocarcinoma and adenoma were comparable to that of the ND group at 4 months (Table 4), suggesting that the rate of tumor formation had slowed at this time point.

**Table 3:** Daily food and inorganic phosphate intake and body weight in K-ras^LA1^ mice fed a normal or high-phosphate diet for 1, 2, or 4 months.

	1 month		2 months		4 months	
	ND	HPD	ND	HPD	ND	HPD
Daily food intake (g/mouse)	2.45 ± 0.62	2.88 ± 2.41	2.85 ± 1.37	3.01 ± 3.23	3.21 ± 2.49	3.56 ± 3.29
Daily Pi consumption (mg/mouse)	7.34 ± 0.62	28.9 ± 0.74***	8.55 ± 1.05	30.1 ± 1.92***	9.63 ± 0.84	35.6 ± 0.82***
Mean weight change (g)	5.34 ± 1.72	5.36 ± 2.98	10.14 ± 2.36	10.79 ± 2.98	12.72 ± 1.08	12.38 ± 1.70

Pi, inorganic phosphate; ND, normal diet; HPD, high-phosphate diet.

Data are shown as mean ± SD (n = 6).

***P < 0.001.

**Table 4:** Tumor incidence in the lungs of K-ras^LA1^ mice fed a normal or high-phosphate diet for 1, 2, or 4 months.

ND (n = 6)	Adenocarcinoma	Adenoma	HPD (n = 6)	Adenocarcinoma	Adenoma
1 month	1	3	1 month	2	9
2 months	3	12	2 months	6	14
4 months	6	14	4 months	7	17

ND, normal diet; HPD, high-phosphate diet.

### Changes in lung ion levels are induced by an HPD

**Fig 2:**
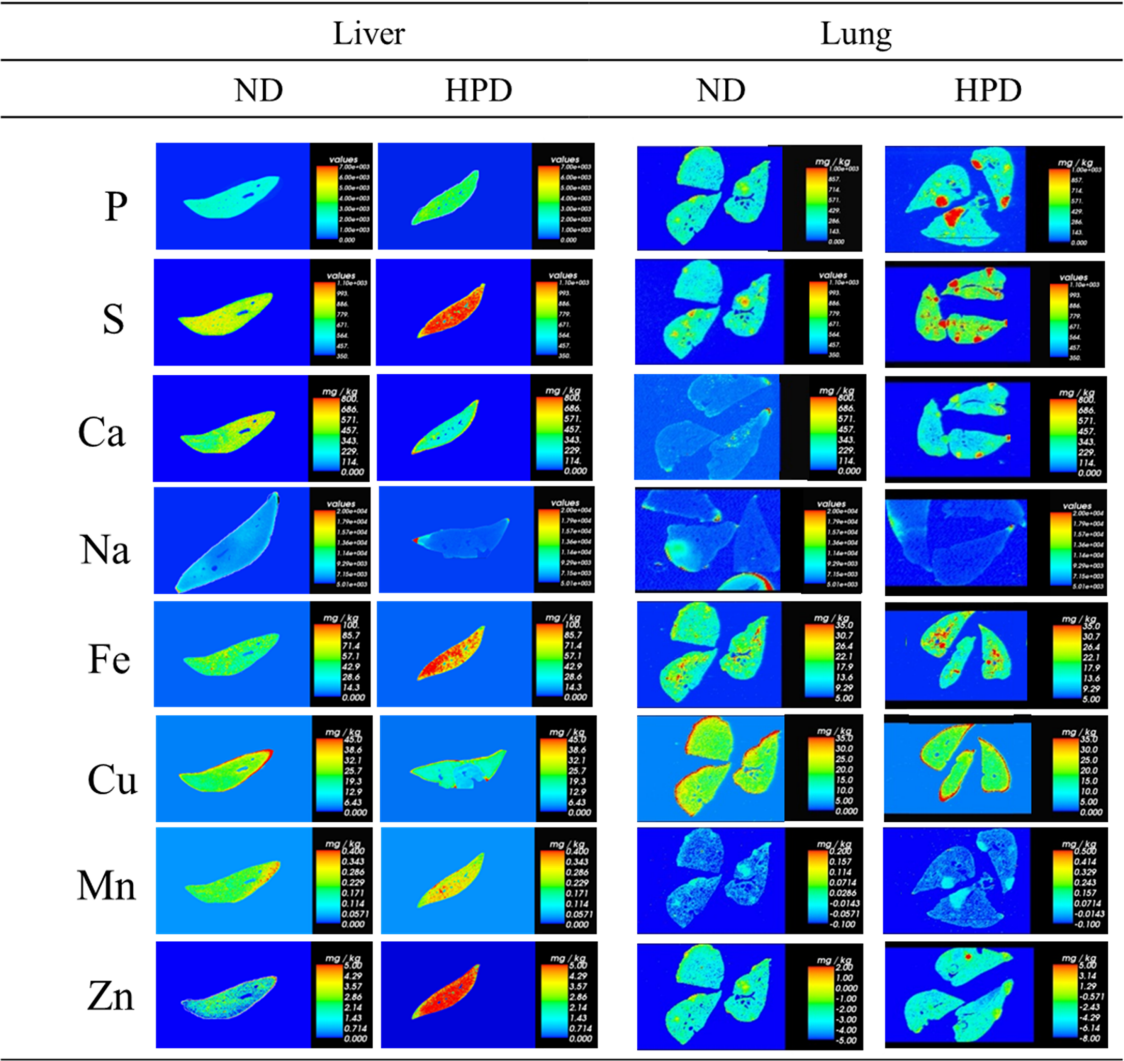
Changes in ion levels in the liver and lungs of K-ras^LA1^ mice provided with an ND or HPD for 4 months. LA-ICP-MS analyses represent mean values from three trials of solution ICP-MS/ICP-OES data (n = 6 per group). The vertical bar in graded color from blue to red represents the ion level from low to high, respectively.

Changes in the levels of various ions were determined by ICP-MS, ICP-OES, and LA-ICP-MS. In contrast to mice in the ND group, markedly higher ion levels were detected in HPD-fed mice: P, S, Fe, Mn, and Zn were elevated in the liver and lungs, and Ca level was also higher in the lungs at 4 months (Table 5 and Fig 2). Differences in lung ion levels between the ND and HPD groups were also detected at 1 and 2 months, however, more vivid difference existed between the ND and HPD group at 4 month. These results imply that a high dietary intake of Pi over a prolonged period alters not only P homeostasis but also the levels of other ions.

**Table 5:** Changes in ion levels in the livers and lungs of K-ras^LA1^ mice fed a normal or high-phosphate diet for 1, 2, or 4 months

	1 month				2 months				4 months			
	Liver		Lung		Liver		Lung		Liver		Lung	
	ND	HPD	ND	HPD	ND	HPD	ND	HPD	ND	HPD	ND	HPD
P (μg/g)	3041.5 ± 112	3334.8 ± 84	2048.6 ± 67	2146.4 ± 59	3371.3 ± 94	3570.6 ± 97	2598.6 ± 67	2724.7 ± 41	2870.9 ± 44	3380.7 ± 58*	2229.8 ± 52	2396.2 ± 39
S (μg/g)	2620.2 ± 76	2908.5 ± 60	n/a	89.4 ± 4.8	2735.2 ± 39	3043.4 ± 89*	2.5 ± 0.08	274.4 ± 8.2**	2330.2 ±48	9222.1 ± 209**	167.6 ± 12.1	308.9 ± 11.9*
Ca (μg/g)	22.9 ± 1.89	47.1 ± 3.29**	22.5 ± 2.93	n/a	26.4 ± 2.12	58.0 ± 3.92*	33.3 ± 1.21	98.8 ± 4.98*	92.3 ± 8.21	63.5 ± 3.65*	60.8 ± 7.21	86.2 ± 5.23*
Na (μg/g)	587.9 ± 23.1	679.3 ± 41	2242.7 ± 98.2	2046.6 ± 89.5	645.7 ± 35.1	544.8 ± 29.3	4909.9 ± 213	2452.6 ± 108**	587.9 ± 29.3	722.4 ± 41.2*	2133.5 ± 93.2	2111.0 ± 49.1
Fe (pg/g)	86186.2 ± 2132	102044.0 ± 3654*	29003.6 ± 872	48475.1 ± 998**	113192.7 ± 3984	96882.5 ± 1894*	63109.7 ± 2132	52227.8 ± 1751	71967.5 ± 1620	179979.0 ± 2178**	59086.9 ± 2100	68112.9 ± 1985*
Mn (pg/g)	685.8 ± 39	554.0 ± 41	n/a	n/a	734.4± 21	614.1± 52	n/a	n/a	480.3 ± 29	665.4 ± 37*	n/a	n/a
Cu (pg/g)	6042.6± 102	7328.3 ± 97*	2695.4 ± 32	2195.7 ± 73**	6682.5 ± 92	6734.2 ± 107	43564.5 ± 325	3413.9 ± 298*	3882.9 ± 42	6586.7 ± 49**	30238.4 ± 121	39087.0 ± 872*
Zn (pg/g)	25118.7 ± 548	26758.8 ± 498	11980.6 ± 198	13108.0 ± 244*	28190.6 ± 319	27771.9 ± 299	14991.7 ± 177	17246.1 ± 142*	29098.7 ± 320	32177.9 ± 211**	29801.9 ± 176	35900.7 ± 189**

ND, normal diet; HPD, high-phosphate diet.

Ion levels were measured by inductively coupled plasma (ICP)-mass spectrometry or ICP-optical emission spectrometry.

Data are presented as mean ± SD (n = 6).

*P < 0.05

**P < 0.01

n/a, not available.

### Pi induces energy production via the TCA cycle in the liver

**Fig 3:**
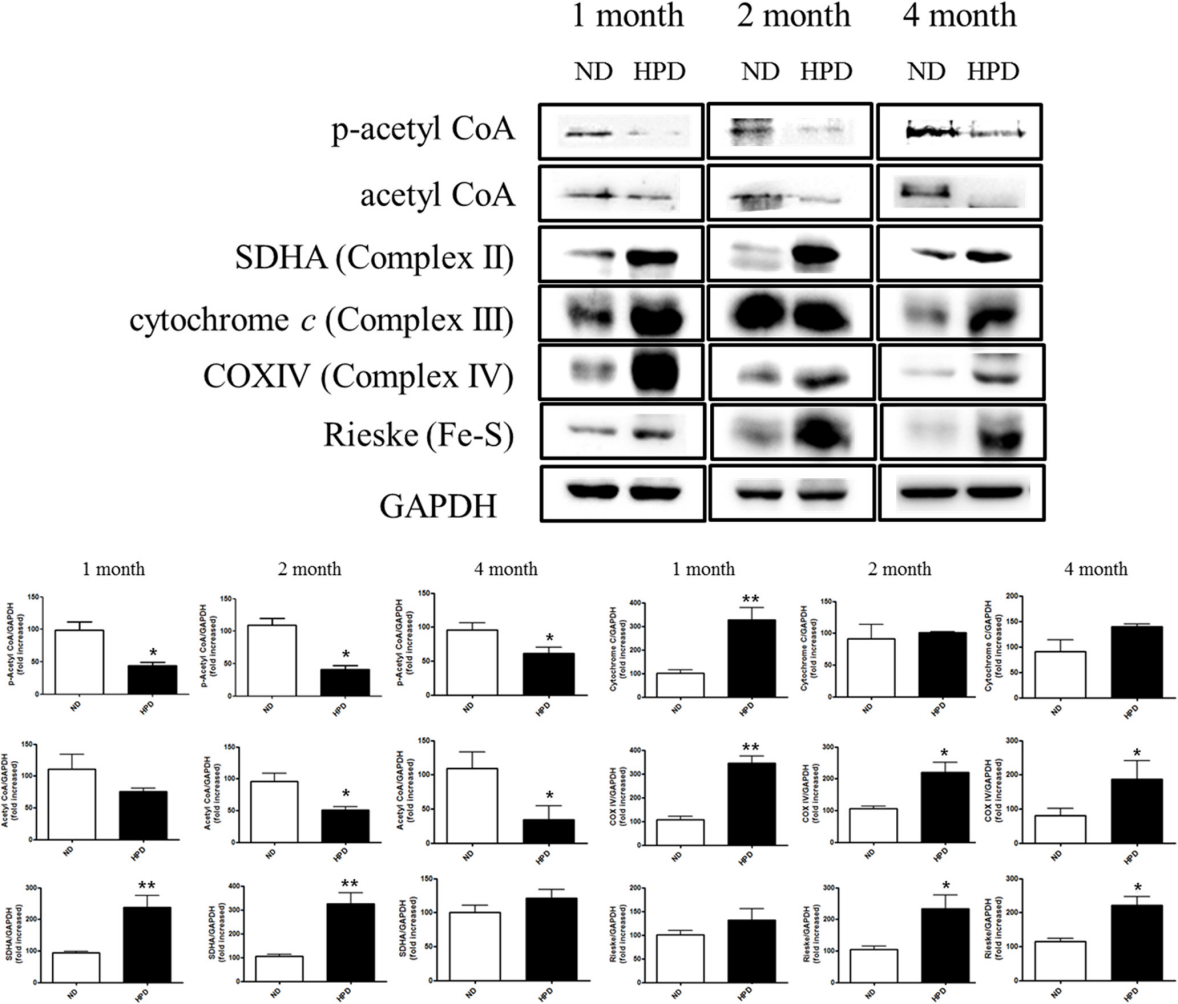
Western blot analysis of cellular metabolism-related proteins. Protein expression of p-acetyl CoA, acetyl CoA, SDHA (complex II), cytochrome c (complex III), COX IV (complex IV), and Rieske (Fe-S complex) relative to glyceraldehyde-3-phosphate dehydrogenase (GAPDH) in liver tissue homogenates of K-ras^LA1^ mice fed an ND or HPD for 1, 2, or 4 months (n = 6 per group), as determined by western blotting and quantified by densitometry. The results are mean ± SD. Error bar represent SD. *p < 0.05; **p < 0.01.

To investigate the effects of an HPD on cellular metabolism, the levels of total and p-acetyl CoA, and complexes II, III, and IV were evaluated by western blotting. A downregulation in the expression of p-acetyl CoA in the liver was observed in the HPD group relative to control mice at 4 months, while acetyl CoA expression was nearly absent (Fig 3). Conversely, the levels of complexes II, III, and IV were upregulated at 1, 2, and 4 months in the HPD group as compared to those in the ND group in the liver. Moreover, the level of the Fe-S Rieske complex was increased in the HPD group relative to the ND group. These results indicate that high Pi intake causes a dysregulation of cellular metabolism, leading to greater ATP production.

### An HPD increases autophagy and epithelial-to-mesenchymal transition in the lungs

**Fig 4:**
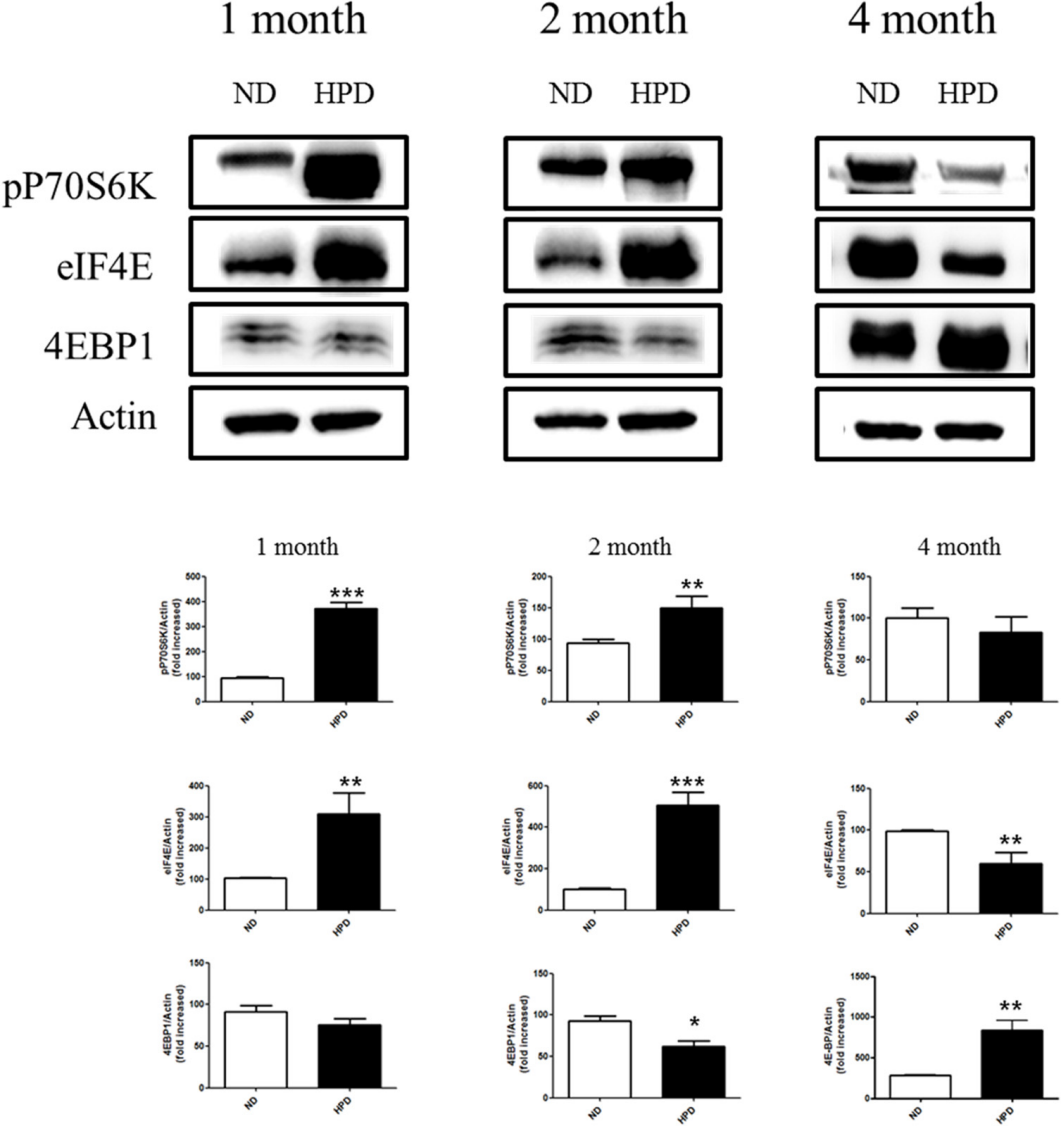
Western blot analysis of EMT- and autophagy-related proteins and qRT-PCR analysis of epithelial marker. (A) Protein expression of the autophagy markers LC3 and ATG5 in lung tissue homogenates as determined by western blotting, using actin as a loading control. (B) LC3 expression (green) in lung tissue sections was assessed by immunocytochemistry. The highest immunoreactivity was observed in the 4-month HPD group relative to the corresponding control (ND) group. Scale bar: 10 μm. (C) Protein expression of the EMT marker N-cadherin in lung tissue homogenates of K-ras^LA1^ mice fed an ND or HPD for 1, 2, or 4 months (n = 6 per group), as determined by western blotting, using actin as a loading control. The results are mean ± standard deviation SD. Error bar represent SD. *p < 0.05; **p < 0.01; ***p < 0.001. (D) qRT-PCR analysis of epithelial markers, E-Cadherin. The results are mean ± standard deviation SD. Error bar represent SD. *p < 0.05; ***p < 0.001.

The previous results indicated that high Pi consumption initially promotes but later inhibits tumorigenesis. To assess whether changes consistent with tumor suppression occur as a result of prolonged high Pi consumption, markers for autophagy and epithelial-to-mesenchymal transition (EMT) were examined. The expression of the autophagosomal markers LC3 and ATG5 was increased in the lungs of HPD-fed mice as compared to ND-fed mice at 4 months, as detected by western blotting (Fig 4A). In lung tissue samples, occasional LC3 signals were observed in the 1- and 2-month HPD groups by immunocytochemistry (data not shown), whereas at 4 months, the fluorescent signal was significantly higher in the HPD than in the ND group (Fig 4B). In addition, the expression of the EMT marker N-cadherin was higher in the HPD than in the ND groups, although the difference between the two dietary conditions was greatest at 4 months (Fig 4C). In qRT-PCR analysis, expression of epithelial marker E-cadherin was lower in the HPD than in the ND groups (Fig 4D). These results suggest that tumor progression is suppressed by high Pi consumption at later stages due to the activation of autophagy and EMT.

### HPD consumption increases protein translation at early stages of tumor development

**Fig 5:**
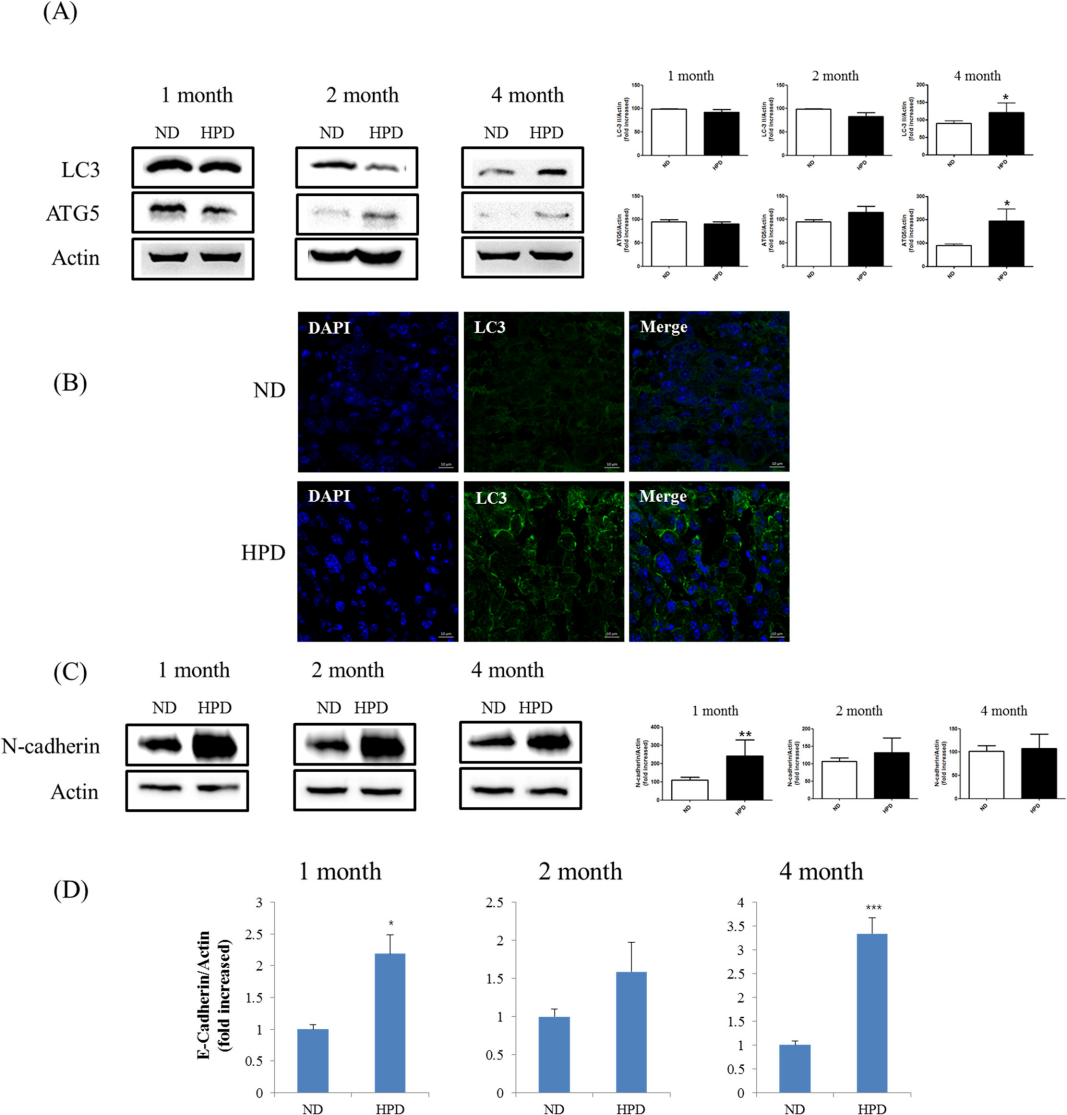
Analysis of protein translation in the lungs of K-ras^LA1^ mice provided with an ND or HPD. The expression of the translation-related proteins p70S6K, eIF4E, p-4E-BP-1/2, and 4E-BP-1/2 in lung tissue homogenates of K-ras^LA1^ mice fed an ND or HPD for 1, 2, or 4 months was determined by western blotting and quantified by densitometry (n = 6 per group) relative to the expression level of actin. The results are mean ± standard deviation SD. Error bar represent SD. *p < 0.05; **p < 0.01; ***p < 0.001.

The mTOR pathway regulates protein translation via eukaryotic inhibition factor 4E binding protein 1 (eIF4E-BP1) phosphorylation and the 70-kDa ribosomal protein S6 kinase (p70S6K), thereby affecting tumor cell proliferation. A significant increase in eIF4E and p70S6K expression was observed in samples from the 1- and 2-month HPD groups relative to the control group (Fig 5). In contrast, the levels of both proteins were decreased at 4 months in the HPD group as compared to the ND group. These data suggest that protein translation was transiently stimulated by Pi during the early stages of tumor development.

### An HPD stimulates cell proliferation and angiogenesis at early stages of tumor development

**Fig 6:**
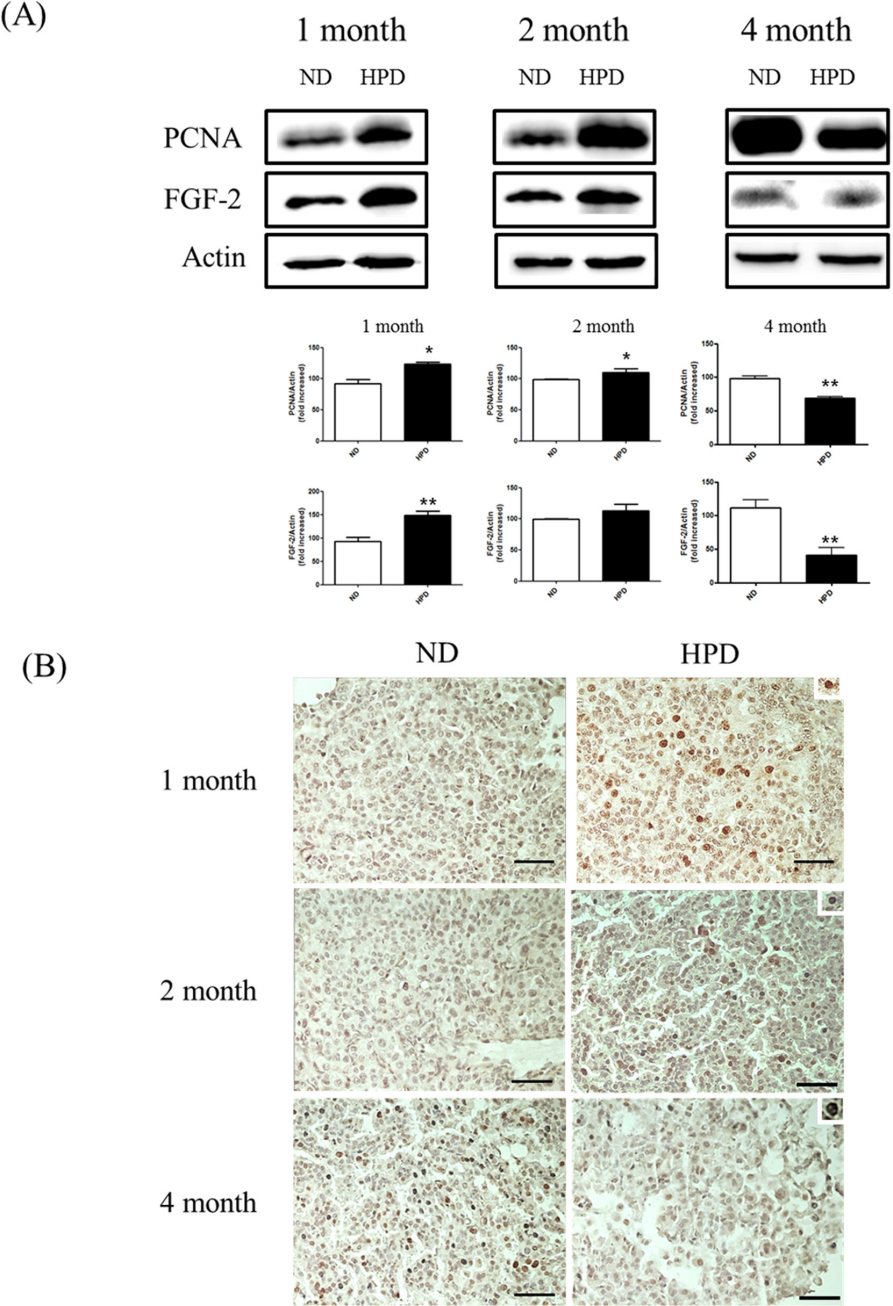
Analysis of proliferation and angiogenesis. Proliferation and angiogenesis were evaluated in the lungs of K-ras^LA1^ mice fed an ND or HPD for 1, 2, or 4 months (n = 6 per group). (A) Expression of proteins associated with proliferation (PCNA) and angiogenesis (FGF-2) in lung tissue homogenates was evaluated by western blotting, using actin as a loading control. (B) PCNA-expressing cells in the tumor region (upper right corner of each panel) were visualized by immunohistochemistry. Scale bar: 20 μm. The results are mean ± standard deviation SD. Error bar represent SD. *p < 0.05; **p < 0.01.

The effect of Pi on tumor progression was also investigated by evaluating cell proliferation and angiogenesis in K-ras^LA1^ mice. The expression levels of PCNA and FGF-2—markers of proliferation and angiogenesis, respectively—in the lungs of mice in the 1- and 2-month HPD groups were increased as compared to those in the corresponding ND groups, as determined by western blotting (Fig 6A). In contrast, a significant decrease in these proteins was observed in the 4-month HPD group. These results were also confirmed by immunohistochemistry, which revealed greater PCNA immunoreactivity in samples from the 1- and 2-month HPD groups than in those from the 4-month HPD group (Fig 6B). These results demonstrate that tumor cell proliferation and angiogenesis were accelerated by Pi at early stages, but that these effects were abolished with prolonged Pi consumption.

### Pi reduces mitochondria-mediated apoptosis in lungs

**Fig 7:**
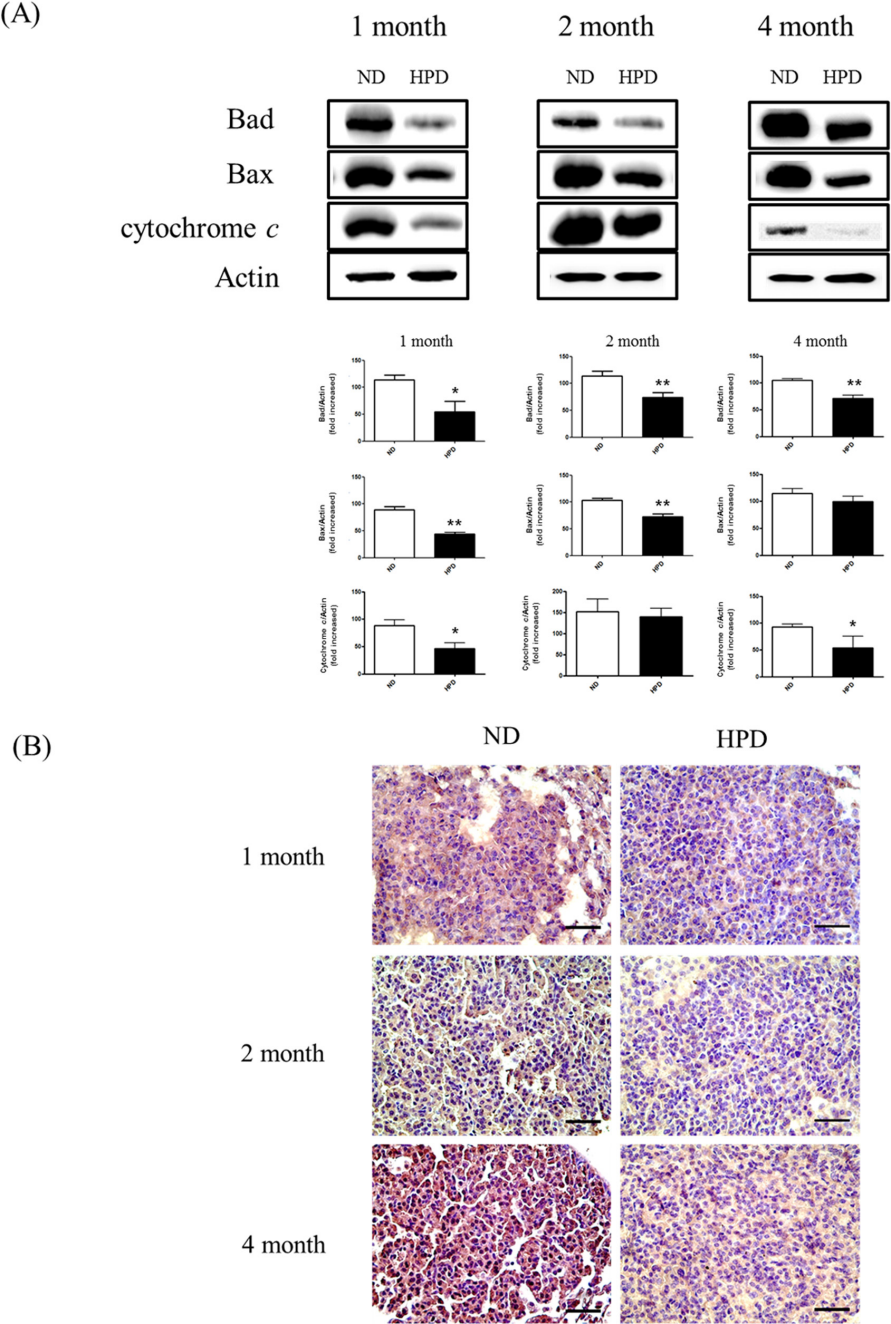
Apoptosis in the lungs of K-ras^LA1^ mice provided with an ND or HPD. Apoptosis in the lungs was analyzed in K-ras^LA1^ mice fed an ND or HPD for 1, 2, or 4 months (n = 6 per group). (A) The expression of the mitochondrial apoptosis-related proteins Bad, Bax, and cytochrome c was evaluated by western blotting of lung tissue homogenates, with actin used as a loading control. (B) Immunohistochemical analysis of caspase-3 expression in the tumor region. Scale bar: 20 μm. The results are mean ± standard deviation SD. Error bar represent SD. *p < 0.05; **p < 0.01.

Evasion of apoptosis is a contributing factor in carcinogenesis, cancer progression, and the acquisition of resistance to therapy. To investigate whether Pi consumption affects mitochondria-mediated apoptosis, the expression levels of Bad, Bax, and cytochrome c proteins in the lung were assessed by western blotting. All three proteins were downregulated in the HPD-fed mice at all time points (Fig 7A). Moreover, caspase-3 activity in the tumor region was also decreased at 1, 2, and 4 months in the HPD group as compared to the ND group (Fig 7B). These results indicate that an HPD can reduce mitochondria-mediated apoptosis in the lungs of K-ras^LA1^ mice.

## Discussion and Conclusion

Neoplastic progression involves a disturbance of the homeostatic control of cell proliferation, cell death, and DNA repair resulting from endogenous factors or exposure to exogenous carcinogenic chemicals [11]. Pi acts as a global signaling molecule that regulates gene expression in fungi, bacteria, and plants, as well as various cell types in animals [12] in processes such as mineral or intermediary metabolism and energy transfer. In addition, Pi is an essential component of phospholipids and nucleotides, and is required for protein phosphorylation [13].

Inhibition of Na-dependent phosphate co-transporter 2b suppressed lung cancer growth; the protein was also found to be highly expressed in stages I and III human lung cancer tissue samples [14]. In a mouse model of lung cancer, high Pi intake has been linked to enhanced cap-dependent translation as well as tumorigenesis via the Akt signaling pathway in the brain, lung, and liver [6,15–17]. However, the effects of prolonged high Pi consumption on pulmonary cancer progression have not been previously reported; this was investigated in the present study in K-ras^LA1^ mice fed an HPD over a period of 1, 2, or 4 months.

The mice consumed a modified version of the AIN-93 diet, which lacks Ca and P. The composition of the pellets was modified based on the AIN-93G diet by replacing the missing elements (for example, by adding sodium phosphate for P) to meet ND (0.5% Ca, 0.3% P) and HPD (0.5% Ca, 1% P) conditions. The daily consumption of Pi was increased while daily food intake was unchanged (Table 3). Moreover, there was no difference in body weight between ND and HPD groups (S1 Fig). Nonetheless, marked changes in the levels of various ions, including P, were observed in the 4-month HPD group, relative to the ND group (Table 5).

The liver is an important organ for digestion as well as xenobiotic metabolism. Blood exiting the stomach and intestines passes through the liver, which breaks down the nutrient components [18]. Since this process involves alterations in ion levels, a quantitative analysis was performed to assess changes in the concentrations of various ions in mice fed an HPD. An analysis of the Fe-S Rieske complex by LA-ICP-MS revealed higher Fe and S levels in the livers of HPD-fed mice than control mice that were likely due to increased cellular metabolism. The P level was also increased in all HPD groups. A higher metal ion intake increases the risk of cancer by stimulating cell proliferation or inhibiting tumor suppressors such as p53. For example, cytoplasmic Ca^2+^ acts as a mitogen [19]. Cancer occurrence has also been linked to increased uptake and decreased efflux of Fe [20]. Moreover, a high Fe^3+^ level activates Fe^3+^-dependent proteins that enhance cell proliferation [21]. Macrophages in the tumor microenvironment may accelerate tumor growth in part by providing tumor cells with Fe [22], while elevated Fe^3+^ levels may increase cancer risk. High Zn^2+^ levels can induce the loss of mitochondrial potential and result in the degradation of Bcl-2 [23]; Zn^2+^ plays a key role in downregulating apoptosis-related genes such as *Bax*, *Bcl-2*, and *survivin*, which leads to tumorigenesis [24]. Damage to Bcl-2 is a causal factor in various cancers, including melanoma, breast, lung, and prostate cancers, and chronic lymphocytic leukemia [25]. These findings along with the results of the present study suggest that high Pi consumption over a long term alters the levels of specific metal ions and can induce tumorigenesis. Additional work is currently underway to determine the precise role of these ions in cancer development and progression.

The TCA cycle is an energy-generating metabolic pathway that begins with the transfer of a two-carbon acetyl group from acetyl-CoA to oxaloacetate [26]. Complexes I–IV play important roles in cellular respiration and ATP production [27]. We found that the basal metabolic rate was higher in the HPD than in the ND group, suggesting that Pi activated the TCA cycle in the liver through rapid consumption of acetyl-CoA. Indeed, an HPD induced the upregulation of electron transport chain-associated proteins such as SDHA, cytochrome c, COX-IV, and Rieske (Fig 3). Most primary and metastatic cancers are characterized by increased glycolysis, which leads to increased glucose consumption [28]. Glycolysis intermediates are used for biosynthesis by cancer cells, which therefore require more ATP than non-dividing cells [29]; cancer cells can also use aerobic glycolysis to metabolize glucose, a phenomenon known as the Warburg effect [28]. A recent study reported that the endoplasmic reticulum enzyme ectonucleoside triphosphate diphosphohydrolase 5 stimulates ATP consumption and favors aerobic glycolysis, thereby promoting cancer cell survival [30]. The results of the present study indicate that high Pi triggers cellular metabolism and leads to greater ATP production.

Autophagy activation is observed under both starvation and hypoxic conditions in the tumor microenvironment [31]. Autophagy promotes cell survival under transient nutrient starvation and growth factor withdrawal [32] in response to low glucose uptake and glycolysis, which leads to a decrease in the rate of protein translation [33]. Under hypoxic conditions, cancer cells display altered metabolic characteristics such as an upregulation of glycolysis and oxidative stress response. Continuous biosynthesis of lactate from glucose under aerobic conditions represents an adaptation to sporadic hypoxic conditions in premalignant lesions and promotes cancer cell dormancy [34], in which cells in the tumor interior enter a state of quiescence triggered by low nutrient availability and hypoxia. Autophagic activity, as evidenced by LC3-II expression, is induced as an adaptive response [35], while the expression of specific EMT proteins is correlated with tumor aggressiveness [36]. In the present study, it was assumed that tumors adapted to the high levels of Pi that prolonged the low-energy metabolic state, thus resulting in tumor cell quiescence in mice in the 4-month HPD group. During metabolic adaptation to high Pi concentration, cancer cells can also develop drug resistance and become more aggressive [37]. Here, it was observed that the expression of the autophagy markers LC3-II and ATG5 as well as that of the EMT marker N-cadherin was elevated in the lungs of mice in the 4-month HPD group (Fig 4C); this finding is consistent with those of recent studies that have shown that the activation of autophagy is correlated with tumor adaptation and a consequent increase in cancer aggressiveness [38].

Another recent study found that the mTOR-Akt pathway was activated in approximately 90% of non-small cell lung cancer cells, which conferred survival and resistance to therapy [39]. The ribosomal protein p70S6K regulates protein translation via the mTOR pathway, and its inactivation suppresses cell growth [40]. In addition, translation and phosphorylation of 4E-BP1, which is expressed in a variety of malignancies, is closely linked to tumor progression. Translation initiation is inhibited by the binding of 4E-BP1 and eIF4E, leading to the recruitment of the translation complex to mRNA [41]. The assembly of the eIF4F complex is completed by the release of hyperphosphorylated 4E-BP1 from eIF4E, which allows translation to proceed [42]. Translation initiation is the rate-limiting step of protein synthesis, and translation rates are determined by eIF4E protein levels [43]. In the present study, pP70S6K and eIF4E were highly expressed in the lungs of mice in the 1- and 2-month HPD groups, suggesting that protein turnover and tumor progression in these mice were higher than that in mice consuming high Pi over a longer period.

Tumorigenesis involves tumor cell proliferation and angiogenesis [44], which can be detected by PCNA and FGF-2 expression, respectively. The present study found an upregulation of both proteins in the lungs of mice in the 1- and 2-month HPD groups; the lower expression levels at 4 months may have been due to cancer cell quiescence induced as a mechanism of adaptation or to autophagy triggered by the hypoxic tumor microenvironment, resulting in aggressive cancer growth.

Apoptosis is a physiological process that removes damaged cellular components and thereby maintains tissue homeostasis [45]. The intrinsic apoptotic pathway is induced by mitochondrial or DNA damage, resulting in the cytoplasmic release of cytochrome c [46] through a pore-like structure in the mitochondrial outer membrane formed by Bax [47]. We showed that the expression of Bad, Bax, cytochrome c, and caspase-3 was downregulated in the HPD group (Fig 7), which suggests that high Pi inhibited apoptosis, resulting in increased cancer cell aggressiveness.

In conclusion, the results of this study demonstrate a homeostatic response to a high Pi level in the lungs. Dietary intake of excess Pi promoted lung cancer progression at early stages; the subsequent decrease in the rate of tumorigenesis was likely due to metabolic adaptation resulting in tumor cell quiescence, which may ultimately lead to greater cancer severity later on. As such, natural and processed foods or food additives containing high levels of Pi should be avoided by lung cancer patients, especially at the early stages of the disease.

## Supporting Information

S1 Fig Body weight of K-ras^LA1^ mice provided with an ND or HPD. Body weight was measured in mice after 1, 2, or 4 months on each diet (n = 6 per group).
(TIF)
