# Supplementary figures and images for: High Inorganic Phosphate Intake Promotes Tumorigenesis at Early Stages in a Mouse Model of Lung Cancer
Source: PLoS One. 2015 Aug 18;10(8):e0135582. doi: 10.1371/journal.pone.0135582 (PMC4540575; doi:10.1371/journal.pone.0135582)

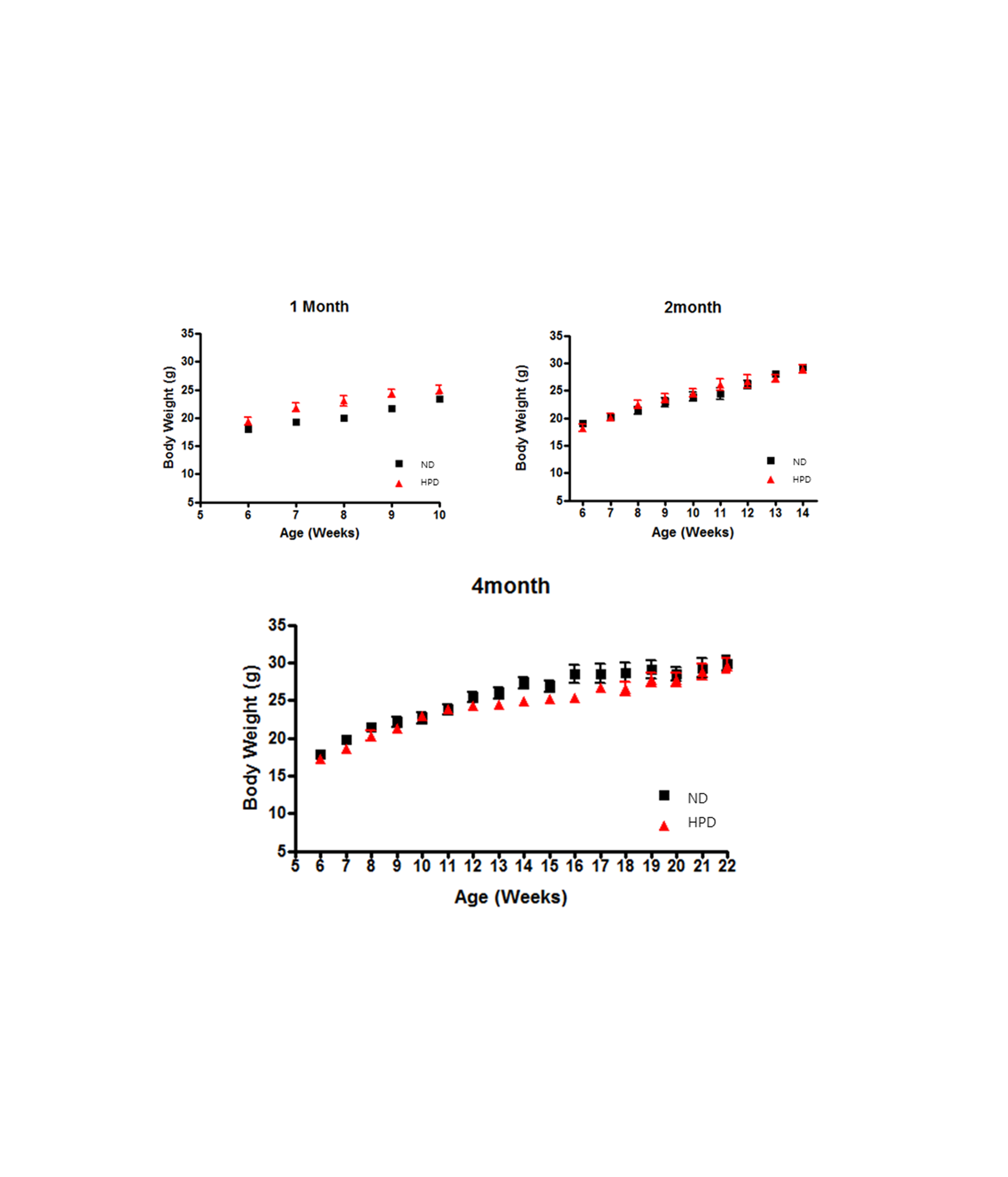

Supplement: S1 Fig — Body weight was measured in mice after 1, 2, or 4 months on each diet (n = 6 per group). (TIF) [file pone.0135582.s001.tif]
